# Local Geographic Variation of Public Services Inequality: Does the Neighborhood Scale Matter?

**DOI:** 10.3390/ijerph13100981

**Published:** 2016-10-01

**Authors:** Chunzhu Wei, Pablo Cabrera-Barona, Thomas Blaschke

**Affiliations:** Department of Geoinformatics—Z_GIS, University of Salzburg, Schillerstrasse 30, Salzburg 5020, Austria; pablo.cabrera-barona@stud.sbg.ac.at (P.C.-B.); Thomas.Blaschke@sbg.ac.at (T.B.)

**Keywords:** inequality, health, public services, neighborhood, scale, PCA, GWPCA

## Abstract

This study aims to explore the effect of the neighborhood scale when estimating public services inequality based on the aggregation of social, environmental, and health-related indicators. Inequality analyses were carried out at three neighborhood scales: the original census blocks and two aggregated neighborhood units generated by the spatial “k”luster analysis by the tree edge removal (SKATER) algorithm and the self-organizing map (SOM) algorithm. Then, we combined a set of health-related public services indicators with the geographically weighted principal components analyses (GWPCA) and the principal components analyses (PCA) to measure the public services inequality across all multi-scale neighborhood units. Finally, a statistical test was applied to evaluate the scale effects in inequality measurements by combining all available field survey data. We chose Quito as the case study area. All of the aggregated neighborhood units performed better than the original census blocks in terms of the social indicators extracted from a field survey. The SKATER and SOM algorithms can help to define the neighborhoods in inequality analyses. Moreover, GWPCA performs better than PCA in multivariate spatial inequality estimation. Understanding the scale effects is essential to sustain a social neighborhood organization, which, in turn, positively affects social determinants of public health and public quality of life.

## 1. Introduction

Analyzing the relationship between the state of the public social services and environmental health has become a major issue for quality of life analysis [1,2]. Public social services and facilities are defined as the urban objects that are designated to fulfill supportive functions related to the health and well-being of the citizens of an urban area [3]. More specifically, the term “public services” is related to the fundamental human rights that a population should have: access to clean water, electricity, education, health care, environmental protection, and so on [4,5]. 

Two key aspects of research on the impact of public services have been identified [6]. The first focuses on inequalities of public service accessibility in neighborhood segregation effects. For instance, a broad range of public accessibility variables, such as access to schools [7], access to food [8,9,10], access to green spaces [11,12,13], access to health services [14,15,16,17], access to recreational services [18,19], etc., play a crucial role in social capital´ definitions, real assets values, and environmental conditions. 

The second focus of public service inequality is concerned with the identification of geographical areas with public health service inequalities and exposure to potential hazards to human health [20,21]. Various relationships between indicators of accessibility to public services and social determinants of public health have been identified in previous studies, including areas within a specified distance to drinking water, the presence of sidewalks and green spaces, and so on, which has also been studied in conjunction with public health surveillance [22,23,24,25,26]. The combination of accessibility-related variables (such as accessibility to local stores, elementary schools, and parks/lakes) has supported health-related behavior such as physical activities [7,27]. The salience of socially-determined factors of public health was also confirmed to account for neighborhood-level variations in a variety of accessibility variables (e.g., accessibility to health facilities and supermarkets) [28,29,30]. These findings have important implications in terms of the increasing demands of multivariate public service analysis. Multivariate accessibility statistics can provide a means of detecting and quantifying truly multidimensional patterns. Such techniques can also contribute towards a solution for multiple comparison problems by controlling accessibility variables in analyzing urban interregional transfer schemes [31,32,33,34]. 

Traditional statistics (e.g., the principal components analyses (PCA), Rank Sum Ratio, Delphi) have been used to estimate the contributions of multiple accessibility variables in describing the characteristics of public services [35,36,37]. Nevertheless, indicators of accessibility to these services may vary depending on the way in which the geographical environment is described. This means traditional multivariate statistics methods, which do not allow the consideration of geographical variations in differentiate multivariate datasets, are not suitable for identifying a combination of characteristics of spatial datasets. The integration of multidimensional indicators, based on their spatial correlations and spatial heterogeneities, is becoming an increasingly important topic in spatial analysis [38,39]. In the field of environmental health, geographically weighted models developed by Brunsdon et al. [40,41] have become the most commonly used methods to address this spatial statistics issue. Especially the geographically weighted principal components analyses (GWPCA), which was implemented by Harris [42], made it possible to assess the spatial variability of data structures by using the robustness and the reliability of related indicators in defining cumulative indices. Nevertheless, this technique has not been widely tested with socioeconomic indicators. Since the public service accessibility indicators may differ significantly between different neighborhood scales, incorporating the GWPCA to construct or spatially represent social health-related indicators can provide an important methodological contribution to evaluating inequality using the potentially multivariate spatial relationships of these indicators.

Some previous studies on public services characterized spatial, environmental, and social indicators on only a certain scale, and they used multiple accessibility variables to evaluate inequality [43,44,45] without considering the sensitivity of these indicators to the neighborhood scale. For example, many studies related to residential neighborhoods and health mainly used administrative or census areas as the geographical units [46,47,48], but whether or not these areas are appropriate or suitable to understand social dynamics is not thoroughly discussed [49,50]. Several studies have also used single variables, like race [51,52], zip codes [53,54], clusters of housing units [55], etc., for neighborhood delineation. However, a subjective neighborhood delineation based on these variables makes it difficult to consider multiple dimensions of the neighborhood’s socioeconomic status, leading to potentially inaccurate analytical outcomes and potentially erroneous recommendations for urban policy makers [27]. In general, specifying the geographical boundaries of neighborhoods can be problematic because neighborhoods vary according to the scales of the observed study regions, and they are subjective in terms of the users or the results of areas where statistical data are available [50,56,57,58]. This uncertainty of the neighborhood scale, which reflects the “cognitive maps of society”, is the well-known modifiable areal unit problem (MAUP) [59,60,61].

A key question regarding the MAUP is how to apply user-controlled neighborhood analysis and flexible geographical units by developing sensible and appropriate zoning systems for particular purposes [60,61,62,63]. Motivated by the uncertainty of MAUP issues about how to construct socially meaningful units for neighborhoods, some studies have attempted to objectively construct practical representations of neighborhoods based on several clusters and regionalization techniques for public health, public service, quality of life, etc. [64,65,66]. For instance, Openshaw [60] and Cockings and Martin [67] proposed the automatic zoning procedure (AZP) to define neighborhood units aggregated from census blocks. This technique used the socioeconomic factors, such as population size [60], deprivation [68], homogeneity of ill-health dynamics intervention policies [67], etc., to address the MAUP issue in delineating socially meaningful neighborhoods. Nevertheless, this procedure is constrained by the requirement of prior knowledge on behalf of the researcher (i.e., the researcher must have prior knowledge). Other urban regionalization methods, like Moran’s I [69], or the symbolic dynamic filtering (SDF) [70,71] are also adopted to identify spatial clusters and spatial outliers of sociologically meaningful neighborhoods. Nevertheless, these methods cannot be used to estimate un-sampled areas, which means that prior knowledge also influences the neighborhood interpolation. The challenge of neighborhood definitions in public social service analysis remains regarding how to objectively exploit the flexibility of using researcher-defined spatial units to appropriately assess the impacts of neighborhood factors.

The self-organizing map (SOM) algorithm [72,73], as an unsupervised learning procedure that explores the topological properties of neighborhood, has been widely used in exploring the homogeneity features of urban evolution [74], the connectivity of the urban street network [75], and city structure characteristics based on remote sensing data [76]. The spatial “k”luster analysis by tree edge removal (SKATER) [77], as a graph-based technique, has the advantages of bringing down computational costs and reducing the sensitivity of clustering procedures by tree edge removal. However, it has rarely been discussed whether these two methodologies are suitable in terms of optimizing the construction of socially homogenous geographical units that most closely represent the reality of the societal situation. Although these methods define the neighborhood boundaries in which cluster methods greatly influence the results of statistical analyses [78], it is still difficult to reach a general conclusion as to which method can produce units that are the most useful for the identification of spatial patterns when identifying the relationship of the variables expressed in those geographical units [77]. Therefore, it is worth paying attention to the comparison of these two well-known methods (SKATER and SOM) in delineating the functional neighborhood and in representing the health-related public services. 

In this study, we aim to explore the neighborhood effects on measuring inequality in public services by considering these services as opportunities to define positive socially determined health outcomes and inequality as the level of access to these services. Based on the analysis of the above-mentioned literature on public service analysis, we selected the multiple accessibility variables that properly represent the public services and are available from the statistical institutes. Firstly, we established multi-scale neighborhood units based on two regionalization approaches: the SOM and SKATER algorithms. These regionalization approaches were based on a composite index of deprivation. Secondly, we applied the PCA and GWPCA to describe the inequalities within these different multi-scale neighborhood systems by analyzing area-based indicators of access to public services. Then, we validated our inequality analyses using individual-based social indicators extracted from a field survey. 

In this study, we discuss the following research questions:

1. Is it appropriate to use SKATER and SOM models to define the socially meaningful neighborhood? What are their sensitivities, their strengths and limitations in social public service inequality analysis?
2. What are the advantages of the GWPCA in measuring inequality in public services within multi-scale neighborhoods compared with the PCA?

## 2. Study Area and Data Collection

### 2.1. Study Area Selection

**Figure 1:**
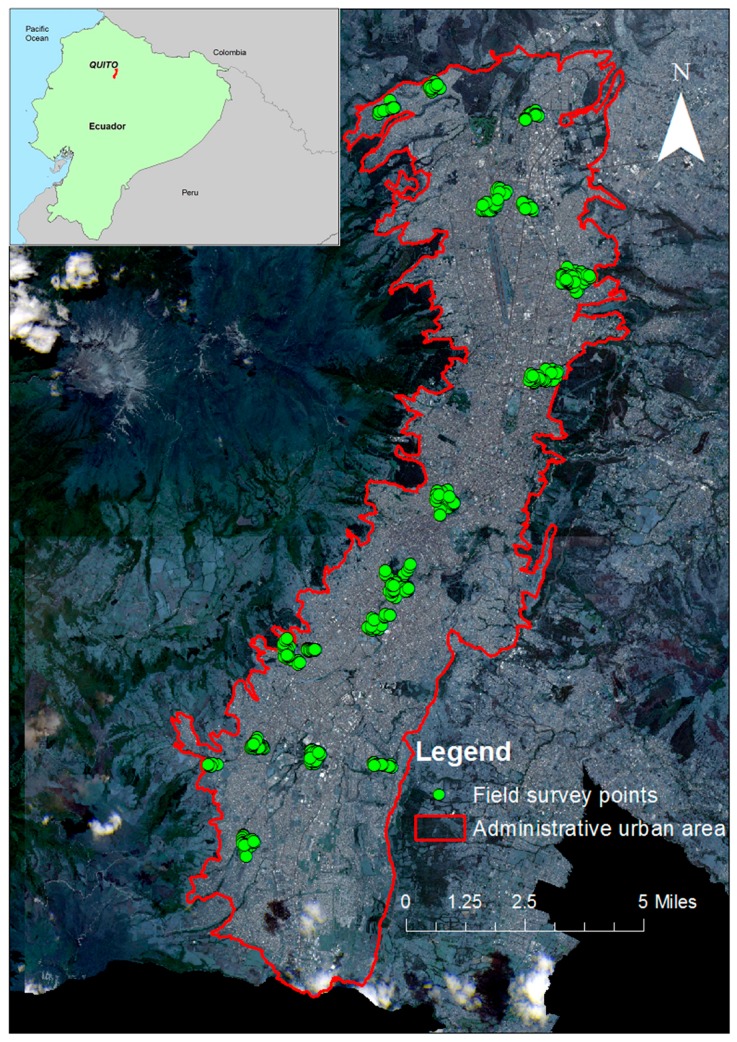
The study area—Quito, Ecuador.

Our study area is the city of Quito, the capital of Ecuador (Figure 1). It is home to more than 1.5 million inhabitants according to the 2010 Ecuadorian Population and Housing Census. The problem of inequality extends across the entire world and Latin America can be considered as the region of the world with the highest inequality in terms of access to services and other socioeconomic variables [79]. In Latin America, large cities have very high accessibilities to services compared to rural areas. Despite this advantage in some areas, deprived areas with unequal access to services can be identified in these cities. The city of Quito is a city with generally good access to services and relatively low deprivation, but it contains some very deprived urban areas with low access to services, i.e., health services [16,80]. 

In Quito, residential land use can be found throughout the city, but it is particularly concentrated in the northern and suburban zones of the city. In the south, land use primarily consists of industrial and commercial zones and low/medium income residential areas. 

### 2.2. Data

The indicators used to characterize public service inequality are shown in Table 1. These indicators were chosen following a rights-based approach of good living [68,81,82]: basic rights, i.e., access to basic services, should be given priority in order to ensure good conditions/prerequisites for quality of life. The chosen indicators are also linked to health-related social dimensions (e.g., education, household conditions) and have been applied to evaluate health inequalities [16,83]. The indicators were extracted from the census, and the results of the applied methods in this study should be applicable to other Latin American cities since they are conceptually similar.

Additionally, analyzing geographical accessibility to urban resources (education, health care services, green areas) allows the identification of spatial and social inequalities with important implications for health planning [84,85]. This kind of accessibility can be defined as the travel impedance for residents in an area to reach certain services and is expressed in terms of distance or time [84,86]. 

Four indicators were calculated using variables of the 2010 Ecuadorian Population and Housing Census and all indicators are based on census blocks (dissemination areas). Four indicators were estimated based on the parameters of *the total number of households in a specific census block* and *the number of households that do not have access to a specific public service*. These indicators are expressed as ratios (households without access to a specific public service vs. total number of households) and include (1) no access to drinking water (NonDri); (2) no access to the sewerage system (NonSew); (3) no access to the public electricity grid (NonEle); and (4) no access to a garbage collection service (NonCol). The variables used to calculate these indicators were extracted from the 2010 Ecuadorian Population and Housing Census. 

Two indicators concerning the accessibility to health services and to educational services were calculated. The geographical accessibility was considered, which, in the case of this study, was calculated as the Euclidean distance to the nearest healthcare service (or educational service) from the centroid of each of the census blocks. This kind of distance has been proven to be a useful measure of spatial accessibility and a proper proxy of travel time [87,88]. The obtained indicators were: limited access to health care services (Dist_H) and limited access to educational services (Dist_E). 

Finally, the indicator limited access to green areas was calculated, which is also considered important for the understanding of urban inequalities and their impact on health [89]. Remote sensing data extracted from 2010 Rapid Eye imagery (Planet Labs, San Francisco, CA, USA) and an analysis of the normalized difference vegetation index (NDVI) based on ARCGIS tool (Esri, Redlands, CA, USA) were used to obtain the indicator of limited access to green areas (Green): the ratio of greenspace in an area unit (census block). 

The field survey conducted as part of this study was carried out in the months of July, August, and October 2014 and encompassed different questions related to quality of life, health, and social dimensions. The survey was conducted throughout the entire study area using a two-stage sampling strategy. In the first stage, ARCGIS tool (Esri, Redlands, CA, USA) was applied to divide the study area into sampling areas (hexagons). Eighteen hexagons were randomly chosen based on the time and financial resources available for the survey. In the second stage, pseudo-random interviews were carried out in each sample area using a door-to-door interview petition to obtain answers in households where people were willing to participate. The number of interviews also depended on the population density and land use of each hexagon. In areas with a higher population and urban cover, more pseudo-random interviews were conducted. 

The survey included questions regarding individual health conditions, social cohesion of the neighborhood, and neighborhood security. The answers to these questions were rated using a 1–5 Likert scale, whereby, for example, in the case of the self-perceived quality of life, a value of 5 indicates that the interviewed person considers themselves to have a very good quality of life. The same logic was applied to the other variables. Four variables were obtained: self-perceived health status (HS), the perceived social cohesion of the neighborhood (NC), the perceived security of the neighborhood (NS), and the self-perceived quality of life (QoL).

The response rate of the survey was 61%. A total of 489 responses was obtained. The random hexagon strategy resulted in obtaining responses in socio-economically distinct neighborhoods. The survey is statistically reliable: 54.6% of the interviewees were female and the sampling margin of error obtained was ±4 (95% level of confidence). 

## 3. Methodology

### 3.1. Workflow

**Figure 2:**
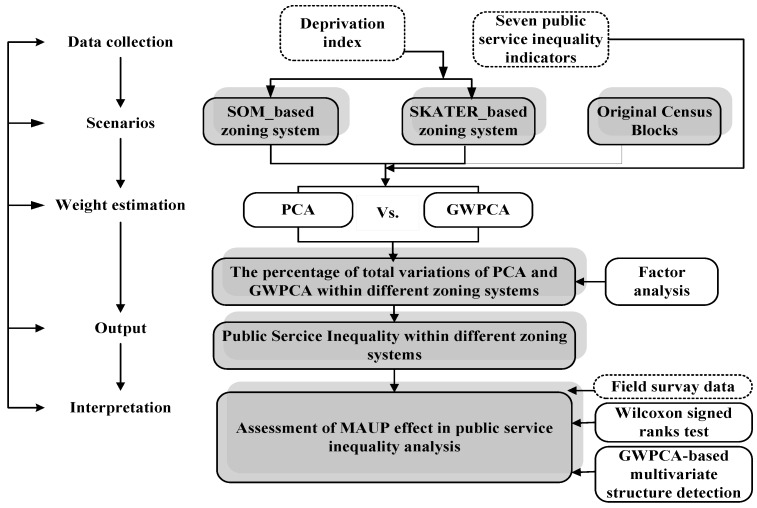
Overall workflow. **White** boxes represent input, **white** boxes with dashed borders represent specific methods, and **grey** boxes represent results. PCA = principal components analyses, GWPCA = geographically weighted principal components analyses.

As shown in Figure 2, the initial step of the workflow was to aggregate two groups of spatial neighborhood units based on the original census blocks. We call each group of neighborhood units a zoning system: each zoning system encompasses areas that are homogenous in terms of deprivation. The homogenization was performed using a deprivation index. The aggregated units based on these two zoning systems were considered as the two types of scenarios to analyze the public service inequality. The original zoning system with around 5000 census blocks was also considered in the analyses. 

The second stage of the study was the evaluation of public services inequality. We applied the GWPCA and PCA separately to integrate the seven indicators of public services inequality (NonDri, NonSew, NonCol, NonEle, Dist_H, Dist_E, Green) into the factor analysis to estimate the weights of these indicators in order to construct measures of public services inequality. These measures were calculated within the different zoning systems. 

The final step was the assessment of MAUP effects on the analysis of public services inequality. This assessment is related to the evaluation of uncertainties in the analysis of inequality. 

### 3.2. Urban Regionalization

#### 3.2.1. The Threshold of Urban Regionalization: The Deprivation Index

Increasing evidence suggests that the urban poverty situation is a key determinant of population health [26]. We thus assumed that people sharing similar social deprivation characteristics have similar access to public services. An urban regionalization can be based on the homogeneity of deprivation. A social deprivation index was thus chosen as the unique threshold variable for the two urban regionalization/clustering methods (SOM and SKATER) applied in this study. A deprivation index has already been developed for our study area [68] and was applied in this study. This index considers several indicators related to health, education, employment, and housing conditions, and has been proven to be related to health-related variables. 

#### 3.2.2. Self-Organizing Maps (SOM)

The SOM method has several advantages: independence from external evaluation functions, recognition of the most meaningful features within vector space, and auto stability of the network [73]. This method was used to automatically map high-dimensional data onto a regular low-dimensional grid. We thus used the SOM to convert complex, nonlinear statistical relationships between the high-dimensional, original objects into simple geometric relationships on a two-dimensional display.

All of the original objects were treated as Kohonen neurons, whereby each neuron has its weight vector indexed with the same attribute dimension, in this case, the deprivation index. Hexagonal grids are the preferred choice for presenting neurons then each node then has six immediate neighbors. After initializing all neurons’ weight vectors, we randomly chose a data point from the training data for the SOM. We then determined its best matching unit (BMU) on the map by measuring the Euclidean distance between the weight vector for each of the Kohonen neurons. The closest Kohonen neuron (i.e., minimum distance) is the chosen neuron. The weights of this chosen neuron and the neurons included within the neighborhood of the BMU are adjusted by the neighborhood function. This learning process consists of selection and adaptation of the synaptic weights until the Kohonen neurons converge to a unique limit. This method has been fully described by Kohonen [73].

#### 3.2.3. Spatial “k”luster Analysis by Tree Edge Removal (SKATER)

SKATER is an efficient regionalization technique that uses minimum spanning trees (MST), which consist of a connected tree with no circuits. It transforms the regionalization problem into an optimal graph partitioning problem [77]. The minimum spanning tree is unique if the costs between any node and all its neighbors are distinct [90]. 

In our research, we created an original MST by establishing a connectivity graph with a set of vertices (V) and a set of edges (L) for the original regions to capture the adjacency relations between objects. The original MST was defined by a Euclidean distance cost d(i, j) with the edge (vi, vj) between the neighborhood objects i and j using their attribute vectors xi and xj. In our MST, the deprivation index represents an unequal attribute to measure the dissimilarity between objects. This index is measured in standard deviation units in order to have comparable scales. Based on the original MST, we used a hierarchical division strategy to prune the MST and obtained a set of spatial clusters. Each resulting cluster will be a tree with all vertices connected and no circuits. The pruning produces a reduced graph that defines a smaller class of possible partitions whose edges join similar categorical attribute areas. In order to maintain consistent levels of the geographical units with the regionalization SOM, we defined the initial node as 40 for the hierarchical division procedure. This procedure has been fully described by AssunÇão et al. [77].

### 3.3. Public Services Inequality Analysis

Our public service inequality analysis began with the identification of multiple accessibility variables linked to data structures or potentially linked to the data structure. The PCA and GWPCA were applied separately to organize these variables. PCA was applied to identify the correlation between the public services indicators and to convert a set of possibly correlated accessibility variables into a set of values of linearly uncorrelated variables called principle components. GWPCA was also used to identify the correlation of the datasets based on their spatial heterogeneity and spatial autocorrelation information. PCA and GWPCA provide the inputs for the factor analysis, which will determine the weights of selected factors in the linear regression model of the public services inequality analysis. 

#### 3.3.1. Principal Components Analysis

PCA is one of the most popular dimensionality reduction methods; it groups together individual collinear indicators to form a composite indicator that captures as much of the information as possible [91]. The main purpose of the PCA application for the seven public services indicators in our research is to explore relationships between them and to identify the highest possible variation in the public service indicator set using the smallest possible number of factors. This methodology has been fully explained by Jolliffe [92].

#### 3.3.2. Geographically Weighted Principal Components

The standard PCA does not consider spatial effects. As an adaptation of the PCA approach, the GWPCA takes the spatial autocorrelation in the spatial process into account [42,93,94]. Incorporating the GWPCA within the same re-design algorithm may provide an improvement for a multivariate spatial process that has distinct non-stationary relationship properties. We used GWPCA to explore the public services inequalities structure through computing a series of localized PCAs within different urban regionalization systems where the local component outputs are mapped, thus permitting a local identification of any change in the structure of the multivariate data. Selecting the kernel weighting function and the size of its bandwidth is the most crucial aspect when implementing the GWPCA algorithm. Therefore, we calibrated our GWPCAs with a bi-square kernel using adaptive bandwidths whose sizes are chosen automatically and objectively via cross-validation. This methodology has been fully explained by Harris et al. [93].

#### 3.3.3. Factor Analysis of Public Services Inequalities

Depending on the correlation structure of the indicator set, it is possible to identify a certain number of latent factors (fewer than the number of individual indicators) to represent the indicator set through a factor analysis [91]. In the factor analysis, the factors that explain more than 10% of the total variance individually and account for 85% of variance overall are initially chosen to estimate the contribution of indicators. Then, each factor that is based on a set of coefficients (the correlation between the individual indicator and the latent factor) is rotated by applying the varimax rotation matrix. The third step is to deal with the construction of weights from the matrix of factor loadings, whereby intermediate components are aggregated by assigning a weight to each indicator according to their proportion of the explained variances in the indicator set. The factor analysis approach has been fully explained by Nardo [91] and Nicoletti [95].

Two measures of public service inequality were calculated by adding the weighted normalized indicators: one measure is based on the weights extracted from the PCA and the other measure is based on the weights extracted from the GWPCA. 

### 3.4. Assessment of MAUP Effects on the Public Service Inequality Analyses

#### 3.4.1. Wilcoxon Signed-Rank Test

In order to validate the measures of public services inequality, data extracted from a field survey, including the variables of individual self-perceived health status (HS), the perceived social cohesion of the neighborhood (NC), the perceived security of the neighborhood (NS), and the individual self-perceived quality of life (QoL), are applied and treated as the ground truth data.

The Wilcoxon signed-rank test is a nonparametric test, analogous to the independent samples *t*-test, and can be used when two independent groups are ordinally scaled [96]. This test is useful to test and understand whether there are median differences when relating the survey data and the results of the public inequality analyses. The *p*-values of the “matched-pair” (of information from the survey data and the public services inequality analysis) were used to determine whether there were statistically significant differences between the underlying distributions of each paired sample. 

#### 3.4.2. The Spatial Heterogeneity of the Set of Public Services Inequality Indicators

In the GWPCA, a specific localized PCA is estimated at a target location, permitting a local identification of any change in structure of a multivariate data set. Harris et al. [93] integrated the GWPCA methodology with robust local Mahalonobis distances (MDs) to outline any change in the structure of a multivariate data set. Therefore, we can use this GWPCA-based detection method to investigate the local scale characteristics of a multivariate composition for (1) checking whether the data structure of the observation is unusual with respect to its close spatial neighborhood; and (2) investigating how the MAUP influences a certain spatial heterogeneity in the structure of the set of public services inequality indicators. Harris et al. [93] have presented the full report on this methodology.

## 4. Results

### 4.1. Visualization of the Variances of Multiple Accessibility Indicators in the Different Zoning Systems

**Figure 3:**
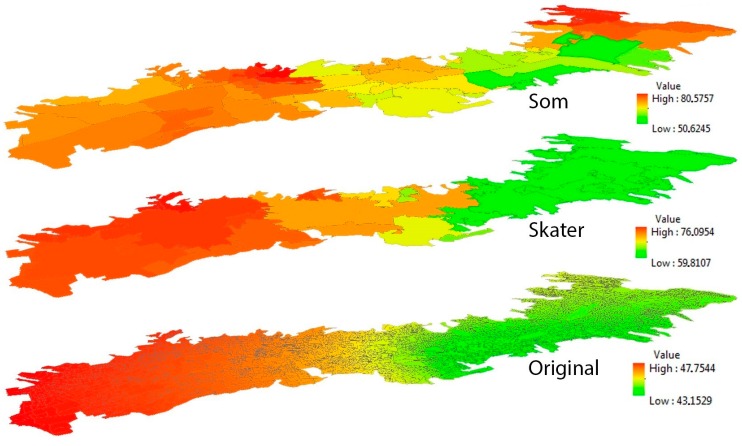
The first principal component of the GWPCA in three zoning systems.

It is important to emphasize that GWPCA reveals local spatial structures in a given multivariate data set when compared with global PCA. Using the global PCA, it was possible to ascertain that the first component loading of seven multiple accessibility indicators encompassed 67.46% and 65.29% of the percentage of the total variances (PTVs) for the SKATER-based and the SOM-based zoning system, respectively, and that these values are higher than the 42.34% PTVs found in the original census blocks. However, it is still difficult to gain insights into the spatial distribution of each composite variable and capture their dimensionality variations in the multivariate dataset. In the GWPCA, these investigations and interpretations all take place locally in each neighborhood unit, that is, the PTVs for the first component in two aggregated zoning systems and in the original census blocks are all summarized at each data location in the study area. As shown in Figure 3, it is appropriate to consider a subset of the components with larger PTVs to contribute to the greater part of data variance. 

The spatial patterns in the PTVs vary significantly between different neighborhood units and different scales. This is especially true for the north of the city where spatial patterns are very sensitive to the scale of the geographical units, ranging from 80.58% to 42.78%. The highest PTVs in the three zoning systems are generally located in the south of the city, while the lowest PTVs are located in the city center. It is also possible to identify the number of components to retain, given some pre-specified threshold of total variance preserved. For example, for a threshold of 85%, only the first principal component is required for the southern regions of Quito in all zoning systems. In many regions of central and northern Quito, the first two (or more) components are required to represent the significant spatial variation of public services indicators values.

### 4.2. The Measures of Public Services Inequalities in the Different Zoning Systems

**Figure 4:**
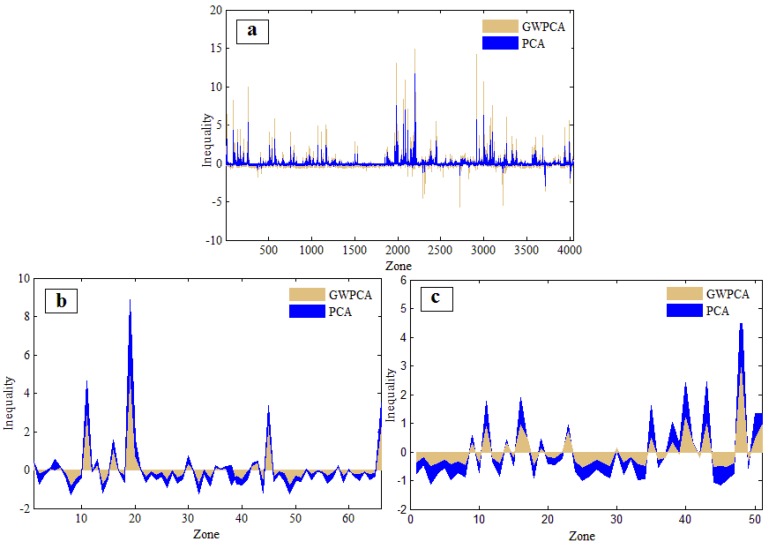
Different public services inequality results based on three different scales of neighborhoods units: (**a**) original census blocks; (**b**) self-organizing map (SOM)_based zoning system; and (**c**) spatial the “k”luster analysis by tree edge removal (SKATER)_based zoning system. The *x*-axes represent the number of neighborhood units in each zoning system, y-axes represent the PCA_ and GWPCA_based measures of public services inequality based on seven accessibility variables (NonDri: no access to drinking water; NonSew: no access to the sewerage system; NonCol: no access to a garbage collection service; NonEle: no access to the public electricity grid; Dist_H: limited access to health care services; Dist_E: limited access to educational services; Green: limited access to green areas).

PCA and GWPCA analysis are used separately to group seven accessibility variables (NonDri, NonSew, NonCol, NonEle, Dist_H, Dist_E, Green) to capture as much of the multivariate information as possible. After weighting the score of each principle component in the PCA- and GWPCA-based groups through factor analysis, these two groups of intermediate principle components are collinearly combined to estimate the final inequality results. This inequality workflow is applied to the original census block system (in Figure 4a), the SKATER-based zoning system (in Figure 4b), and the SOM-based zoning system (in Figure 4c).

It is worth noting that there are similar patterns of public services inequality based on the PCA- and the GWPCA-based measurements for all three zoning systems (shown in Figure 4). Nevertheless, for aggregated-level zoning systems (including the SOM- and SKATER-based zoning systems), the PCA-based measures of inequality are generally a little higher than the GWPCA-based measures (Figure 4b,c). However, an opposite trend appears in the case of the original zoning system (Figure 4a). This means that the spatial heterogeneity of the seven accessibility variables is stronger than the variance decomposition of the dataset in the aggregated-level zoning systems compared to the original census block system. Therefore, it is crucial to account for a certain spatial heterogeneity and autocorrelation characteristics in the multivariate data analysis to achieve multi-scale neighborhood units.

**Figure 5:**
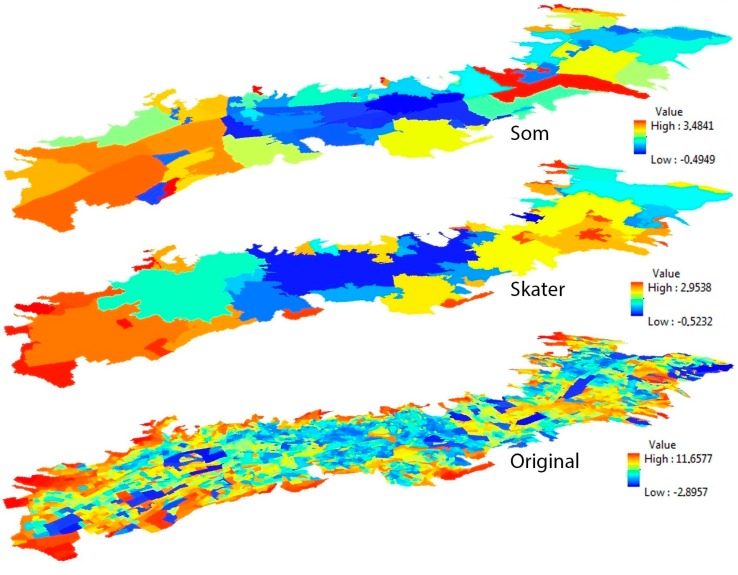
Spatial visualization of the GWPCA-based public services inequality in three different zoning systems: (**a**) SOM-based zoning system; (**b**) SKATER-based zoning system; and (**c**) original census block.

The definition of neighborhood also has a significant influence on the public service inequality estimation. As shown in Figure 5a–c, there are spatial variations in the GWPCA-based public services inequality across different zoning systems. The original zoning system shows specific variations across the city, while—not surprisingly—both aggregated-level zoning systems tend to generalize the levels of public services inequality in larger areas. However, a general trend can be identified: There are peripheral areas of the city in all zoning systems (e.g., areas located in the extreme southeast and the extreme northwest) with the highest values of public services inequality. These areas correspond to the most deprived neighborhoods of the city [68,80]. Another common spatial characteristic is the presence of low values of public services inequality in central and central-northern areas of the city. These areas correspond to the historical downtown (central areas) and to the modern downtown (central-northern areas). 

### 4.3. Assessment of MAUP Effects in the Public Service Inequality Analyses

#### 4.3.1. Wilcoxon Signed-Rank Test 

We used the field survey indicators, namely self-perceive health status (HS), the perceived social cohesion of the neighborhood (NC), the perceived security of the neighborhood (NS), and the self-perceived quality of life (QoL) to validate our public service inequality assessment. As shown in Table 2, more than 75% of paired-samples between the PCA-based measures of public services inequality and the field survey variables indicate statistically significant differences in the three zoning systems. However, differences between the paired-samples between GWPCA-based measures of public services inequality and the field survey variables decrease to 42% within the three zoning systems. 

In the case of the GWPCA-based measures of public services inequality, levels of public service inequality vary significantly between the different neighbourhoods and zoning systems. Table 2 shows that all the field survey indicators are in accordance with the measures of inequality expressed in the SKATER zoning system. The distributions of NC and NS also show no important differences between the field survey and the group of public services inequality in the SOM zoning system. In the case of the census blocks system, only the HS variable shows no obvious difference compared to the field results. 

Therefore, the neighborhood units based on two computer-aggregated zoning systems correspond well to the neighborhood cohesion and neighborhood security; the neighborhood units in the SKATER zoning system especially coincide with the human perception of health-status and quality of life.

#### 4.3.2. The Spatial Heterogeneity of the Set of Public Services Inequality Indicators

To highlight the spatial heterogeneity of the set of public services accessibility indicators, we chose two representative areas of Quito to demonstrate the local outlier of their multivariate structure separately, namely the historical downtown or old city center, and the modern downtown or new city center. Multivariate structure analysis was applied to all the public services accessibility indicators: no access to drinking water (NonDri), no access to the sewerage system (NonSew), no access to the public electricity grid (NonEle), no access to the garbage collection service (NonCol), limited access to health care services (Dist_H), limited access to educational services (Dist_E), and limited access to green areas (Green).

**Figure 6:**
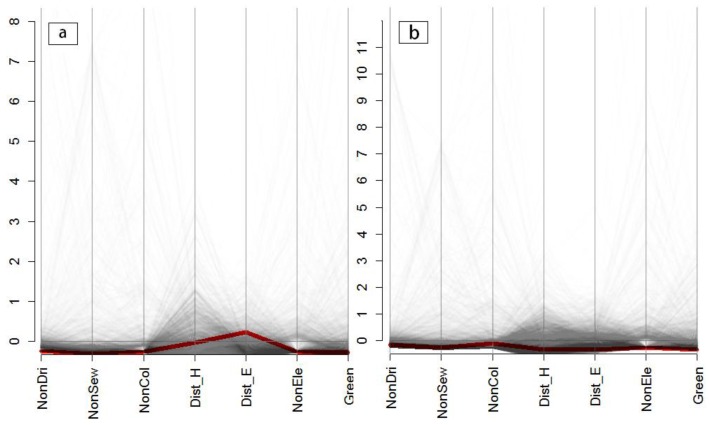
Local outliner detection for the public services accessibility indicators for the original census blocks zoning system: (**a**) for the area of the new city center; and (**b**) for the area of the old city center; *x*-axes represent the seven accessibility variables (NonDri, NonSew, NonCol, NonEle, Dist_H, Dist_E, Green); and *y*-axes represent the re-scaled spatial structures of seven accessibility variables.

**Figure 7:**
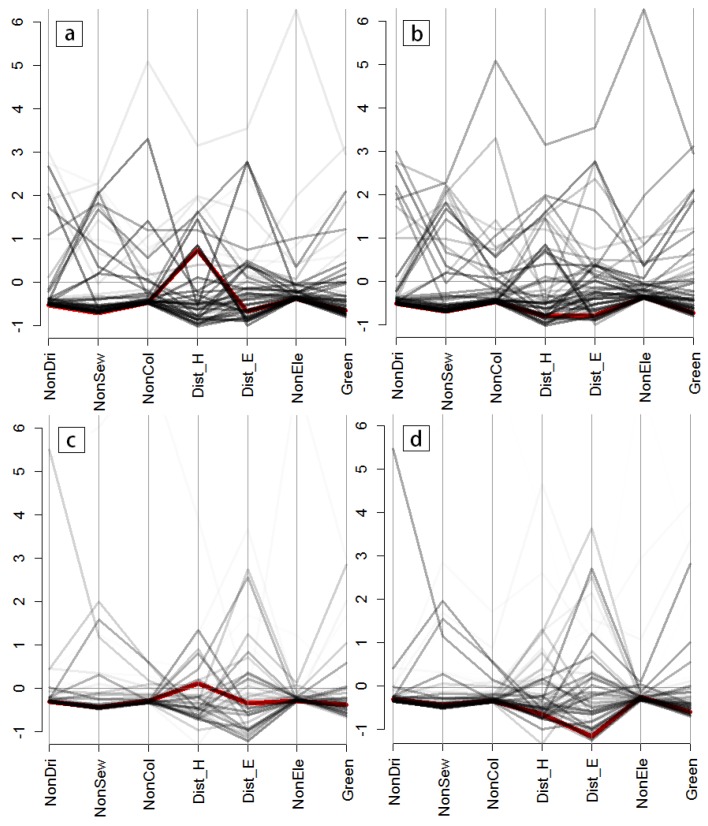
Local outliner detection for the public services accessibility indicators: (**a**) SKATER_based zoning system for the new city center area; (**b**) SKATER-based zoning system for the area of the old city center; (**c**) SOM_based zoning system for the new city center area; and (**d**) SOM_based zoning system for the area of the old city center; *x*-axes represent the seven accessibility variables (NonDri, NonSew, NonCol, NonEle, Dist_H, Dist_E, Green); and *y*-axes represent the re-scaled spatial structures of seven accessibility variables.

The spatial heterogeneities in the structure of the indicators within the three zoning systems are summarized in Figure 6 and Figure 7. Every indicator is re-scaled and represented by a parallel vertical axis. The line depicting the multivariate structure of the chosen areas (the old city center and the new city center) is colored red, while all other lines (in black) are shown as the multivariate dataset structure in the neighborhood areas, with increasing levels of transparency according to their geographical distances from the chosen areas. Transparency levels are weighted via a bi-square kernel, where lines for the most distant sites appear fully transparent.

In the original census blocks (Figure 6), the local spatial structures of accessibility indicators in the old city center (Figure 6b) tend to be similar to their neighborhoods, thus implying that the MAUP effects will not have a significant influence on the inequality analysis at a certain scale at this site. In contrast, the multivariate indicators’ structures (Figure 6a) in the new city center show significant differences to their neighborhoods at a large scale, suggesting that there is a high degree of uncertainty and sensitivity in the spatial statistical analysis at the new city center when considering the scale issues. The same conclusion is reached in a comparison of the spatial structures of the accessibility indicators in the census blocks zoning system (Figure 6) and the two other aggregated-level zoning systems (Figure 7). There are marked spatial structure differences between structure features and local outliers in the original census block and the two aggregated zoning systems in the new city center (Figure 6a vs. Figure 7a,c), but not in the area of the old city center. 

We thus conclude that the spatial heterogeneity of the dataset derived by GWPCA allows us to evaluate the uncertainty of the MAUP effect in accessibility indicators analysis to some extent, and the aggregated zoning procedures based on the SOM and the SKATER methods perform well in clustering neighborhoods with similar public service capabilities. 

## 5. Discussion

The exploration of functional neighborhood units for the optimization of health-related public services is the potential of this study. The two aggregated zoning systems created in this study enable delineating functional neighborhoods that are in accordance with the reality of the social and economic characteristics of Quito. The statistical test of these three zoning systems confirms the previous study and the conclusion that, if properly carried out, regionalization approaches can produce spatial entities that are more useful for spatial pattern analyses than the original data set [68]. Furthermore, the establishment of multi-scale neighborhood units based on the degree of deprivation is a first attempt to explore the spatial representativeness of the MAUP effect on measures of public services inequality. Our results show that there are close relationships between the status of urban deprivation and the health-related public services inequality in Quito, and the deprivation index thus has the ability to act as a threshold in the urban regionalization to describe the functional neighborhoods for public services analysis.

We have shown that the social and health-related indicators used to calculate public services inequality represent a degree of spatial heterogeneity across multi-scale geographical units, not considering the MAUP effects in spatial dataset analyses may generate misleading results for decision-makers. Our results show that even though the PCA is a statistical technique widely used in many studies, it ignores spatial effects and spatial characteristics in multivariate data sets, and the PCA-based inequality results do not perform better than the GWPCA-based inequality results in relation to the field survey data. The GWPCA-based inequality results clearly show the spatial heterogeneity of data structures and regional differences in public services analysis. The GWPCA also enables full use to be made of geographically weighted correlation coefficients in a certain observation location to identify the MAUP effect. For example, the old city center of Quito does not show the obvious differences in the structure of the multivariate data set with its neighborhood at a large scale, meaning that the public inequality analysis at this site is not likely to be biased by scale and zoning effects at a large scale. In other words, the GWPCA is valuable for investigating the correlations of each indicator with the locally derived components and showing the indicators that differ most significantly compared to those observed in the surrounding neighborhoods.

A better identification of changes in the variables *self-perceived quality of life*, *the self-perceived health-status*, and *the perceived neighborhood cohesion* and *security* was possible in the two zoning systems created. Nevertheless, different clustering procedures largely depend on the structural features of the original blocks and on the capability of different regionalization methods. For example, SKATER can only produce a good-quality partition in a highly efficient manner if the original area has high homogeneity characteristics and is sensitive to the choice of the partition trees, which significantly influence the quality of the resulting partitions. SOM is an unsupervised learning algorithm and works well to improve spatial correlations of the data set, but lack of data or extraneous data will create randomness in the groupings. 

The resolution and the quality of the data are also important criteria when designing new zoning systems because the regionalization approaches largely depend on the characteristics of the aggregated data set. How these approaches best integrate a set of social, economic, or environmental indicators into homogeneous contiguous regions is a question that needs to be further discussed and investigated in future research.

This study focused on indicators, which were based on census variables and distances to services. Such indicators can be obtained for most regions of the world. Therefore, the methodology developed in this study can be applied to other case studies while different methods and techniques can be used for the construction of other, locally relevant indicators. For instance, in the case of accessibility to health care services, indicators based on gravity models and services catchment areas may be used. The Euclidean distances used can easily be adapted to local needs or other measures of spatial accessibility. However, we consider it important to also conceive accessibility as a multidimensional measure, which includes not only geographical variables, but also social, cultural, and economic variables. Future research may apply our methodology using multi-dimensional measures of accessibility. 

We are also aware that the threshold for the partition in the regionalization algorithms is at risk of being subjective. The use of thresholds is only suitable to optimize a particular statistic or model, thus providing a map-based visualization of the interaction between data, model or statistic, and the level of aggregation. We recognize that there is no decisive way to define the most suitable scale because the definition of geographical unit scale or neighborhood scale depends on the combination of characteristics in many different disciplines. Therefore, the complex urban system, including environmental conditions, neighborhoods, and residential effects, would result in varying public services based on the scales of different geographical units. The multi-scale zoning systems provided in this study offer the possibility but not the solution to explore the MAUP effect in the evaluation of public services inequality. 

Generally, the principal difficulty concerning zone-design is to choose the most suitable design for a particular purpose. The two zoning systems created for our study are homogenous scenarios in terms of deprivation, in which measures of public services inequality were calculated and then evaluated from a MAUP perspective. However, we consider this study as a first step in order to evaluate suitable scales to analyze inequality. The explanation of inequality related to access public services or any other kind of inequality requires different models and approaches such as regression analyses. 

Areas located in Quito´s periphery are areas with high deprivation [68,80]. Our results confirm this conclusion and provide more evidence of the fact that areas face higher levels of deprivation when their access to specific public services is limited. The public services inequality evidenced in the original census blocks can support healthcare experts and urban planners in developing and applying specific and local-based policies, while the SOM and SKATER zoning systems may support the identification of larger areas of the city that require more general urban policies. The results of (the use of) these two zoning systems have created scenarios that demonstrate the importance of considering geographical variations in the analysis of public services inequality in Quito. 

## 6. Conclusions

We proposed a framework to analyze public services inequality considering seven indicators and applying PCA and GWPCA. These analyses were performed in three zoning systems: one was the original census block system of the study area, and the other two zoning systems were based on the homogenization of deprivation values using the SOM and SKATER algorithms. The variations of the indicators across multi-scale neighborhoods allowed a better understanding and interpretation of public services inequality considering a geographical perspective.

This methodology can be used to better monitor inequalities in a given territory and to partially overcome the scale issue related to data aggregation. It is also an attempt to explore the spatial representativeness of a data set: the spatial heterogeneities and spatial autocorrelations may have important implications for results and conclusions used by decision makers and planners in the functional delineation of neighborhoods. 

Our study also reflects the well-recognized difficulty of defining a neighborhood, both at the conceptual and the operational level. We propose the consideration of additional variables in future studies, e.g., socioeconomic indicators to represent potential threats that can enhance social inequality, to further explore the implications of neighborhood on the basis on this methodology. Our results encourage further research to develop concepts and methods for analyzing social and environmental factors by considering different spatial and non-spatial hierarchical levels. Urban regionalization can be viewed as a dynamic process: subjective boundaries mainly depend on the location of the residents and characteristics of public services. We need neighborhood definitions that relate to public services and reflect social determinants of public health, but also serve as measures of residential segregation—localities that share similar sentiments, traditions, and history. The concept of neighborhood and its definition are, therefore, central to residential segregation and residential location choice analysis. The combination of urban regionalization methods with the analysis of social variables within multi-scale neighborhood zoning systems is crucial to supporting actions in reducing social inequalities and to supporting a local needs-centered urban planning.

## Figures and Tables

**Table 1:** Variable description and their expected effects on public services inequality.

Indicators (Ratios)	Variables	Sign ^a^
No Access to Drinking Water (NonDri)	Number of Households without Access to the Public System of Drinking Water; Total Household Number	+
No Access to Sewerage System (NonSew)	Number of Households without Access to the Sewerage System; Total Household Number	+
No Access to the Public Electricity Grid (NonEle)	Number of Households without Access to the Public Electricity Grid; Total Household Number	+
No Access to Garbage Collection Service (NonCol)	Number of Households without Service of Garbage Collection; Total Household Number	+
Limited Access to Health Care Services (Dist_H)	Distance to the nearest Healthcare Service	+
Limited Access to Educational Services (Dist_E)	Distance to the nearest Educational Service	+
Limited Access to Green Areas (Green)	Ratio of Greenspace in an Area Unit	−

^a^ Sign indicates whether high indicator values increase (+) or decrease (−) access to a specific service, that is to say, increase or decrease inequality.

**Table 2:** The *p*-value of all matched samples in the Wilcoxon signed ranks-test.

Zoning System	Number	HS	NC	NS	QoL
Census Blocks (GWPCA_PSI)	142	0.059 *	0.012	0.000	0.000
Census Blocks (PCA_PSI)	142	0.000	0.633 *	0.000	0.000
SOM (GWPCA_PSI)	19	0.003	0.821 *	0.134 *	0.003
SOM (PCA_PSI)	19	0.001	0.512 *	0.014	0.001
SKATER (GWPCA_PSI)	14	0.058 *	0.065 *	0.714*	0.058 *
SKATER (PCA_PSI)	14	0.001	0.627 *	0.013	0.001

Census Blocks represents the original census blocks zoning system, SOM represents the zoning system based on the self-organizing map method and SKATER represents the zoning system based on the spatial “k”luster analysis by tree edge removal. GWPCA_PSI represents the public service inequality results based on geographically weighted principal components analyses and PCA_PSI represents the public service inequality results based on principal components analyses. Number represents the number of polygons in each zoning system that contains survey data. Asymptotic significances are displayed. Tested at the 0.05 level, the hypothesis that there is no difference between the estimation results and the field survey data will be rejected if the *p*-value is smaller than 0.05, * represents there is no the differences between the estimation results and the certain field survey parameter within the certain zoning system.
